# Organizational health literacy in Austria—policy developments and results from a pilot assessment in primary care

**DOI:** 10.3389/fpubh.2026.1802212

**Published:** 2026-05-14

**Authors:** Christina Dietscher, Christa Straßmayr, Lisa Gugglberger, Julia Eder, Denise Schütze

**Affiliations:** 1 Department Non-Communicable Diseases and Mental Health, Austrian Ministry of Health, Vienna, Austria; 2 Competence Centre Health Promotion and Healthcare, Austrian National Public Health Institute, Vienna, Austria

**Keywords:** health literacy policies, health literacy responsiveness, health literate organizations, OHL-PHC, organizational health literacy, primary care, self-assessment

## Abstract

Within the health literacy (HL) discourse, the development and spread of organizational health literacy (OHL) reflects a shift from viewing HL as an individual trait to a systems-level responsibility, as organizational structures can hinder or support individual HL. By improving these structures, the HL of targeted populations can be systematically strengthened, helping to mitigate socio-economic effects on health outcomes and promoting health equity. Austria began work on OHL in the 2010s, earlier than many European countries. Inspired by the US Institute of Medicine’s “10 attributes of health literate organizations” a concept and self-assessment tool for hospitals was published in 2013. Since then, OHL has become a key element of Austria’s national HL strategy and a focus of the Austrian Health Literacy Alliance. This work has led to the development of tools and supported the integration of OHL into the national healthcare system, for example through HL-related questions in routine patient surveys that promote organizational learning. For health equity, primary care is important not least through supporting the HL of people in the context of basic healthcare provision. In Austria, HL support is legally defined as an organizational responsibility of primary care settings. Against this background, the article provides background information on OHL developments in Austria, focuses on recent data and results from piloting an OHL assessment tool in primary care settings in the context of the European Joint Action PreventNCD, and discusses potential learnings for the Austrian Health Literacy Alliance and for supporting HL and equity through primary care.

## 1 Introduction

Health literacy (HL) is increasingly being discussed as a concept that can help mitigate some of the negative effects of the social determinants of health. However, HL itself comes along with a social gradient (1, 2). Organizations cannot change the education or the socio-economic background of their clients and patients, but they can adapt their structures and processes to the needs of their target groups according to the concept of universal precautions (3), an approach that has become known as organizational health literacy (OHL). This marks a shift from understanding HL as an individual feature and responsibility to a systems and structural level: organizational and system-level features are identified as either hindering or supporting individual HL, making HL—or the mitigation of low HL—a systems and organizational responsibility. In established OHL concepts, both user-oriented dimensions, such as access, communication, and navigation, and organizational or structural dimensions, such as leadership, staff development, and the anchoring of OHL in institutional processes, are integral components (4–6). By changing OHL related organizational features, the HL of large groups of targeted audiences can be systematically and effectively supported (4, 6–9).

In Austria, work on OHL was taken up in the aftermath of Austria’s participation in the first European HL survey HLS-EU (10). Data pointed to the fact that Austrians had more difficulties in a number of HL related tasks than other Europeans. This inspired a national health goal to improve population HL (11). Since there were plausible hypotheses about the systemic and organizational causes of the differences between HL in Austria and other European countries, improvements in OHL were considered important to achieve better population HL from the beginning.

Inspired by the “10 attributes of health literate organizations” of the US Institute of Medicine, Austria developed and published 2013 one of the first European OHL tools – the V-HLO (12) which comprised a concept, a self- assessment tool and an improvement toolbox for hospitals. Based on this work, OHL became one of the permanent focus areas of the Austrian Health Literacy Alliance, which was founded in 2015. There is now a national working group on OHL in Austria. Based on the V-HLO, the group adapted OHL tools to other types of organizations including primary care and non-health care settings such as extracurricular youth work and workplaces. OHL was also mainstreamed into core processes of the national healthcare system. For example, regular national inpatient surveys in Austria today comprise a set of HL related questions to support organizational learning in hospitals.

Primary healthcare soon became a focus of the activities because of its relevance for health equity in the population at large, in the context of basic healthcare provision. While Austria still has a large number of traditional GP offices, more structured primary care units (PCUs) are being implemented throughout the country. Besides GPs, they also employ nurses, physiotherapists, social workers, and other health professionals. In 2025, there were 100 PCUs across Austria (13). The support of patient HL has been defined as an organizational responsibility of PCUs in the Austrian National Health Plan, which is the basic national tool for the planning of healthcare provision (14). This is in line with the WHO Declaration of Astana on Primary Health Care (15) which encourages the improvement of HL through reliable information: “We will promote health literacy and work to satisfy the expectations of individuals and communities for reliable information about health. We will support people in acquiring the knowledge, skills and resources needed to maintain their health or the health of those for whom they care, guided by health professionals.” PCUs, so far, are the only healthcare organization in Austria which are legally mandated to practice HL, which resulted in a need to support PCU staff in taking up HL through training and tools.

With its national experiences, Austria is also aiming to inspire R&D around OHL on an international level. The country hosts the coordination center of the WHO Action Network on Measuring Population and Organizational Health Literacy (M-POHL) (16, 17) and initiated work on OHL in this network. Within the European Union’s Joint Action PreventNCD (18), Austria coordinates a sub-task to further develop and disseminate OHL in the European Union with the aim to reduce inequalities in health (19). In the following, we are reporting on piloting an OHL tool for primary care within this Joint Action.

## 2 Pilot-assessment of OHL in primary care

### 2.1 Methods and materials

#### 2.1.1 The OHL-PHC tool

**Figure 1:**
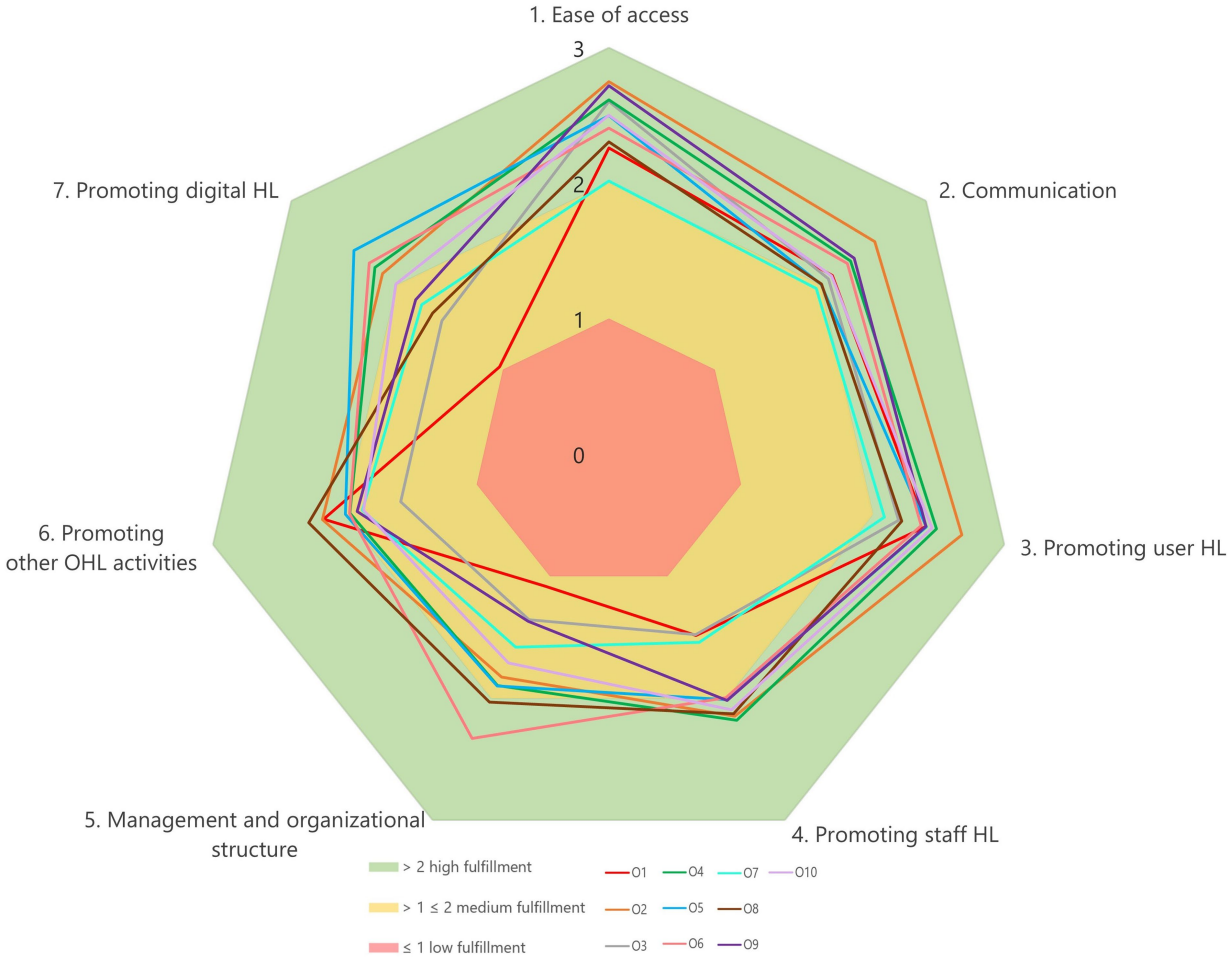
The average degree of fulfillment (mean) for standards 1–7 for each of the 10 primary care units (O1 to O10). Score range 0–3. Shaded areas indicate fulfillment categories.

**Figure 2:**
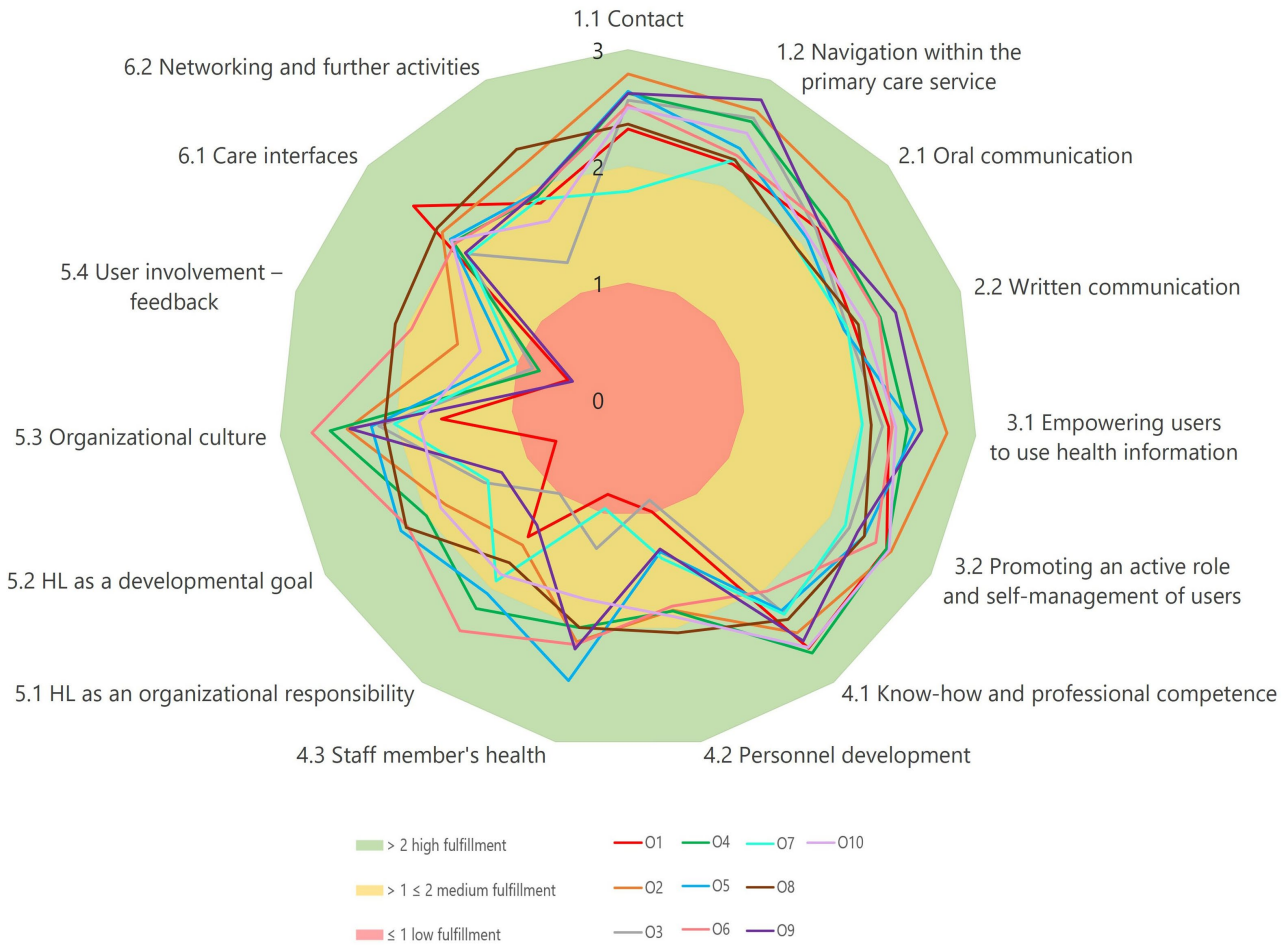
The average degree of fulfillment (mean) for sub-standards 1.1–6.2 of each of the 10 primary care units (O1 to O10). Score range 0–3. Shaded areas indicate fulfillment categories.

The International Self-Assessment Tool for Organizational Health Literacy in Primary Health Care Services (OHL-PHC) (20), was used in this explorative study. The tool was translated into German and culturally adapted to the Austrian health care context following M-POHL’s standardized procedure (19). The OHL-PHC comprises seven standards (see Figure 1), 15 sub-standards (see Figure 2) and 70 indicators or measurable elements. Indicators can be rated according to the degree of fulfillment: to a very large extent (76–100%), to a large extent (51–75%), to some extent (26–50%), or to a small extent/not fulfilled (0–25%). In addition, there is a category for indicators not applicable for the organization. The tool includes step-by-step guidance on how to perform the self-assessment process (20).

#### 2.1.2 Data collection

PCUs were invited via mail to participate in a pilot self-assessment process. To adequately capture the different perspectives within each PCU, the tool instructions recommended nominating a coordinator and involving an interdisciplinary and interhierarchical team of at least five people. This composition was intended to ensure that the self-assessment captures diverse organizational perspectives relevant to OHL. A total of ten PCUs – including one specialized pediatric PCU and one Municipal Primary Health Care Center serving as a low-threshold, multilingual contact point for vulnerable groups with a multidisciplinary health care team – volunteered to participate in the study. The sample was a convenience sample, recruited on a first-come, first-serve basis according to the interest expressed by the PCUs, with the goal of including a total of 10 PCUs for the piloting. The participating PCUs represented structural and regional diversity, ranging from village/rural to metropolitan settings, and varied in size from 12 to 56 staff members; the number of full-time equivalents ranged from 6 to 21. Altogether 106 participants took part in the individual assessments (range 6 to 14 participants per PCU) and 124 (range 6 to 24 participants per PCU) in the joint assessments. Participants included a broad mix of professional groups: general practitioners, nurses, assistants/reception staff, social workers, physiotherapists, psychologists, dietitians, occupational therapists, chronic care staff, and trainees.

The self-assessment consisted of two main parts. First, participants completed individual assessments using the tool which was provided online. The researchers provided an overview of the anonymized results from the individual assessments to the coordinators. Thereafter, a joint assessment was conducted during a team meeting, moderated by the coordinators. In the joint assessment the results of the individual assessments were discussed – especially indicators with divergent ratings – and indicators could be re-rated based on consensus. Furthermore, areas for improvement were identified, and next steps towards becoming a health-literate health care organization were defined. The research team trained the coordinators on the self-assessment tool and the process. The self-assessments were conducted from June to November 2025.

After completion, the researchers conducted semi-structured telephone interviews with the coordinators to obtain their experiences on the self-assessment and feedback on the overall assessment process.

#### 2.1.3 Data analysis

Based on the results of the joint assessments, descriptive data analysis was performed. Means for each standard and substandard were calculated and categorized into three groups, indicating: (i) areas of strengths (mean > 2.0); (ii) areas of the intermediate stage needing attention (mean > 1.0 and ≤ 2.0); and (iii) areas of weaknesses (needing attention, mean ≤ 1.0). Standard deviations were categorized, indicating consensus level: (i) high (*sd* < 0.75), (ii) medium (≥ 0.75 and < 1.0), and (iii) low (*sd* ≥ 1.0). This categorization was successfully applied in a feasibility study using the OHL-Hos tool (21).

The study was conducted as an explorative quality assessment project and adhered to the established ethical principles of research, including informed consent, voluntary participation, confidentiality, and data protection. No personal health data were collected, and all data were analyzed in anonymized form. No ethical approval was required for such a study.

## 3 Results

### 3.1 Average degree of fulfillment of standards and sub-standards

The analysis of the mean values for the degree of fulfillment (from 0 indicating no or very low fulfillment to 3 indicating a very large extent of fulfillment) of the seven OHL standards in each of the 10 PCUs (O1–O10) shows varying levels of implementation (Figure 1).

Overall performance is moderate to high in *Ease of access* and *Communication*, with most organizations’ ratings within the high-fulfillment band. *Promoting user HL* also trends positively, albeit with greater dispersion. Lower and more varying results are visible for *Promoting digital HL* and *Promoting other OHL activities*, indicating these as common development needs. *Management and organizational structure* and *Promoting staff HL* show heterogeneity across organizations, suggesting uneven institutionalization of OHL. One PCU (O1) shows low values across several dimensions, which indicates uneven institutionalization of OHL.

Overall, the results from the standards suggest that while user-oriented measures are well established, organizational and structural aspects of HL require further development.

An analysis of the sub-standards allows for a more detailed picture: The means in the degree of fulfillment (scores 0–3) for each of the 10 PCUs in the Sub-Standards 1.1–6.2 are shown in Figure 2. Most PCUs demonstrate high fulfillment in *Contact* (1.1) and *Navigation* (1.2), as well as *Oral* and *Written communication* (2.1, 2.2). Sub-standards related to *User empowerment* (3.1, 3.2) and *Personnel development* (4.1) show medium fulfillment, indicating partial integration of HL principles into patient engagement and personnel development to improve professional HL. The lowest scores appear in *User involvement – feedback* (5.4) and *Networking and further activities* (6.2), highlighting limited mechanisms for participatory governance and external collaboration. Similarly, *Staff members’ health* (4.3) and *HL as organizational responsibility* (5.1) show considerable variability, suggesting these areas lack systematic implementation. While some PCUs achieve consistently high scores across multiple domains, others remain in the low range for several dimensions. This heterogeneity points to uneven institutionalization of OHL practices.

### 3.2 Feedback from the coordinators

Coordinators found the self-assessment tool valuable for identifying their OHL strengths and improvement areas. The training on the self-assessment process and tool was considered essential for a successful implementation. They described the self-assessment process as instrumental in enhancing staff understanding of HL and fostering a more comprehensive perspective on the topic. Joint assessments enabled reflection, discussion, and occasional re-rating of indicators, fostering team consensus. The participatory approach was appreciated, promoting engagement and internal communication. All coordinators reported that the self-assessments led to enhanced staff understanding of OHL, as well as new ideas or concrete plans for OHL improvement interventions, such as enhancing website accessibility, improving signage, expanding multilingual resources, and providing staff training. Lack of best-practice examples to guide organizations on improvement interventions was a concern of the coordinators. The coordinators emphasized that OHL improvement is a continuous process requiring dedicated teams and organizational commitment.

## 4 Discussion

This study yields three main findings: First, the self-assessment tool proved to be feasible and appropriate for Austrian PCUs and shows potential to contribute to reducing health inequalities. Second, the results highlight specific areas that appear particularly challenging for Austrian PCUs to address. Third, while some supportive structures for OHL already exist in Austria, important forms of support remain underdeveloped or absent. The following paragraphs discuss the three main findings in more detail.

First, the feasibility and perceived usefulness of the self-assessment tool are examined. Self-assessment tools have proven useful in supporting monitoring, self-reflection and continuous quality improvement activities (21–23). This study showed that the piloted OHL-PHC tool was perceived as feasible and appropriate in terms of time and effort, although coordinating joint assessments posed minor organizational challenges. External guidance was perceived as helpful, as reported in other studies (24–26). OHL, specifically the self-assessment thereof, can contribute to advancing health equity through systematic identification and reduction of structural barriers that affect all patients but importantly also vulnerable groups. By identifying challenges in communication, navigation, or decision-making processes, self-assessments can make inequities visible at the systems level, which is a prerequisite for equity-oriented action. By specifically identifying and helping patients with low HL, vulnerable and socially disadvantaged groups can benefit.

The second main finding relates to the content of the self-assessment results and highlights specific domains in which Austrian PCUs appear to face challenges in implementing OHL. Data as an interesting starting point for supporting organizational learning around OHL in PCUs in Austria point to areas where primary care organizations need more training and tools to support better performance.

This pilot study shows that across all the participating PCUs, user-oriented standards such as *Ease of access*, *Communication*, and *Promotion of user HL* were well established. These findings may partly reflect the fact that Austrian PCUs are legally mandated to implement health literacy practices and health promotion activities, which likely contributes to the comparatively high levels of fulfillment observed in user-oriented standards. In contrast, organizational and structural dimensions showed moderate to low fulfillment. In particular, the integration of HL into management structures, systematic user feedback mechanisms, personnel development, and digital HL revealed considerable room for improvement. Digital inclusion emerged as a persistent challenge, especially regarding accessibility for users with limited digital skills.

Two pilot studies showed very similar results, one using the V-HLO (12) which was conducted in Austria, and another one using the OHL-Hos tool (21), the international and further developed version of the V-HLO (27), which included but was not limited to Austrian data: The greatest need for development was identified in the areas of participation (which corresponds to part of Sub-standard 5.4 *User involvement – feedback*), organizational anchoring of HL (which corresponds to Sub-standard 5.1. *HL as an organizational responsibility*) and employee training for health-literate communication (which corresponds to Sub-standard 4.2. *Personnel development*). The studies concluded that these three areas could all benefit significantly from more supportive frameworks in the overarching national healthcare system, such as legal requirements, training curricula or funding mechanisms. A recent review supports these conclusions by emphasizing that improving OHL requires systemic efforts and participatory approaches (9).

Third, the findings are discussed in relation to existing and missing support structures at the systems level. The pilot study clearly identified structured external support as crucial for the initial phases and when implementing OHL interventions. This includes training and guidance on OHL and the self-assessment tool, as well as ongoing capacity-building opportunities tailored to different professional groups. Practical resources such as promising practice examples and implementation guides can support the translation of assessment results into action. At the policy level, legal mandates, incentives, funding opportunities, and sharing aggregated data can encourage the uptake and integration of OHL.

In Austria, policy-level initiatives already exist to support both the development HL (11) in the population and the strengthening of OHL (14). The Austrian Health Literacy Alliance promotes networking, collaboration, and knowledge transfer, coordinates measures across political and social sectors and develops trainings and tools. In addition, the Austrian Primary Health Care Platform (13) supports HL and health promotion implementation in primary care by organizing lectures, networking and offering information. Given that Sub-standard 5.4. *User involvement – feedback* scored particularly low – a pattern that already emerged with previous studies of OHL in hospitals – the participatory involvement of patients appears to be a developmental area in the Austrian healthcare system.

While the findings provide valuable insights, some limitations need to be acknowledged. The significance of this study is limited by opportunistic sampling. Of the 100 Austrian PCUs, only 10 participated in the study, meaning that the difficulties observed might underestimate the difficulties in the system overall. Furthermore, it should be noted that the self-assessment reflects staff perspectives only. Although this is inherent to organizational self-assessment approaches, it may systematically underrepresent barriers faced by users, particularly in areas such as navigation, participation, and access. Consequently, some challenges experienced by patients may not have been fully captured. The involvement and participation of users/patients could therefore be expected to provide further important input for the development of OHL.

## 5 Conclusion

Compared to other European countries, Austrian health policy started to support OHL relatively early. However, there is still limited evidence on how PCUs are performing in terms of OHL implementation. This pilot study is a step towards closing this gap and demonstrates that self-assessment of OHL is a feasible and meaningful approach for Austrian PCUs. Based on other studies, the findings indicate that self-assessment can support organizational learning and contribute to advancing health equity by making structural barriers visible, particularly those affecting vulnerable patient groups. While user-oriented aspects of care are largely well developed, challenges remain in organizational anchoring, staff development, digital inclusion, and participatory feedback mechanisms.

Beyond pointing out OHL strengths and weaknesses, these results have several implications: At the organizational level, self-assessment can serve as a structured entry point for reflection, prioritization, and continuous quality improvement, helping PCUs to translate abstract HL concepts into concrete actions. At the professional level, the identified deficits underscore the need for targeted training in health-literate communication, digital inclusion, and user involvement, tailored to different staff roles. At the system and policy levels, the findings highlight the importance of sustained external support, including guidance, incentives, and accessible implementation resources, to enable organizations to act on assessment results. Further systematic data collection is needed to better identify OHL challenges across primary care settings and to inform targeted support measures. In this context, the provision and dissemination of promising-practice examples and implementation models for OHL in primary care may facilitate learning and accelerate improvement efforts. Embedding OHL into existing quality frameworks and monitoring systems may further strengthen its uptake and sustainability.

While these findings are grounded in the Austrian primary care context, several insights are likely transferable beyond this setting. In particular, the feasibility and added value of structured, team-based OHL self-assessment, as well as the recurring pattern of stronger performance in user-oriented domains and development needs in organizational anchoring, staff development, digital inclusion, and participatory feedback, are consistent with findings from other settings and may be relevant across health systems. However, the specific implementation pathways and speed of institutionalization and, to some extent, the level of fulfillment achieved in certain domains depend on national legal mandates, funding arrangements, and coordination structures. Transfer to other contexts therefore requires adaptation to local governance and policy conditions.

Overall, strengthening OHL through systematic self-assessment, combined with comprehensive data collection and analysis, practical support, and enabling system-level conditions, has the potential to improve the accessibility, quality, and equity of primary care in Austria. Future research should examine long-term implementation processes, the impact of self-assessment–guided interventions, and the perspectives of patients, particularly those from vulnerable groups.

## Data Availability

The datasets presented in this article are not readily available because of the privacy of the participants and the participating organizations. Requests to access the datasets should be directed to not available.

## Author disclaimer

This project (GA – 101128023) is co-funded by the European Union. Views and opinions expressed are however those of the author(s) only and do not necessarily reflect those of the European Union or European Health and Digital Executive Agency (HaDEA). Neither the European Union nor HaDEA can be held responsible for them. Views and opinions expressed are however, those of the authors only and do not necessarily reflect those of the European Union or European Health and Digital Executive Agency (HaDEA). Neither the European Union nor HaDEA can be held responsible for them.
